# Role of the Receptor for Advanced Glycation End Products in Heat Stress-Induced Endothelial Hyperpermeability in Acute Lung Injury

**DOI:** 10.3389/fphys.2020.01087

**Published:** 2020-10-07

**Authors:** Gengbiao Zhou, Zhenfeng Chen, Jieyu Li, Xiaotong Guo, Kaiwen Qin, Jiaqi Luo, Jiaqing Hu, Qiaobing Huang, Lei Su, Xiaohua Guo, Qiulin Xu

**Affiliations:** ^1^Department of Emergency and Critical Medicine, Guangdong Provincial People’s Hospital, Guangdong Academy of Medical Science, Guangzhou, China; ^2^Department of Pathophysiology, Guangdong Provincial Key Laboratory of Shock and Microcirculation, School of Basic Medical Sciences, Southern Medical University, Guangzhou, China; ^3^The Second Affiliated Hospital of Guangzhou University of Chinese Medicine, Guangzhou, China; ^4^Department of Intensive Medicine, General Hospital of Southern Theatre Command of PLA, Guangzhou, China

**Keywords:** RAGE, hyperpermeability, heat stress, acute lung injury, C-Jun, HSF1, MAPK

## Abstract

**Objective:**

To study the role of the receptor for advanced glycation end products (RAGE) in endothelial barrier dysfunction induced by heat stress, to further explore the signal pathway by which RAGE contributes to heat-induced endothelia response, and thereby find a novel target for the clinical treatment of ALI (acute lung injury) induced by heatstroke.

**Methods:**

This study established the animal model of heatstroke using RAGE knockout mice. We observed the role of RAGE in acute lung injury induced by heatstroke in mice by evaluating the leukocytes, neutrophils, and protein concentration in BALF (Bronchoalveolar lavage fluids), lung wet/dry ratio, histopathological changes, and the morphological ultrastructure of lung tissue and arterial blood gas analysis. To further study the mechanism, we established a heat stress model of HUVEC and concentrated on the role of RAGE and its signal pathway in the endothelial barrier dysfunction induced by heat stress, measuring Transendothelial electrical resistance (TEER) and western blot.

**Results:**

RAGE played a key role in acute lung injury induced by heatstroke in mice. The mechanism C-Jun is located in the promoter region of the RAGE gene. C-Jun increased the RAGE protein expression while HSF1 suppressed RAGE protein expression. The overexpressed RAGE protein then increased HUVEC monolayer permeability by activating ERK and P38 MAPK under heat stress.

**Conclusion:**

This study indicates the critical role of RAGE in heat stress-induced endothelial hyperpermeability in acute lung injury and suggests that RAGE could be a potential therapeutic target in protecting patients against acute lung injury induced by heatstroke.

## Introduction

Heatstroke is a life-threatening disease characterized by elevated body temperature, usually above 40°C, and neurological dysfunction (Bouchama and Knochel, 2002). Despite immediate cooling and fluid resuscitation, some heatstroke patients deteriorate and progress to multiple organ dysfunction, with mortality reaching 30%. Moreover, more than 30% of the survivors are subjected to long-term sequelae such as permanent central nervous system damage (Yang et al., 2009). Clinically, according to a recent survey, more than three-quarters of the studied heatstroke patients produced multi-organ dysfunction, with the most common dysfunction being respiratory failure (Varghese et al., 2005). An inflammatory cascade reaction develops and strengthens, as a variety of inflammatory cells activate and release inflammatory mediators in response to endogenous or environmental heat, activating ever-increasing numbers of inflammatory mediators or cytokines, which causes increasing damage to the human body. A key treatment for acute lung injury induced by heatstroke is therefore to inhibit this excessive inflammatory reaction (Bouchama and Knochel, 2002).

Our previous study, along with other literature, has demonstrated that vascular endothelial permeability is elevated under heat exposure (Sharma and Cervós-Navarro, 1990; Ng et al., 2004). Endothelial cells play an important role in maintaining the stability of microvascular permeability. Dysfunction of endothelial cell morphology, a disorder that affects the dynamic equilibrium between the intercellular adhesion junction and cytoskeletal tension, along with a contraction of endothelial cells accompanied by a broadening of the intercellular gap, destroys the barrier function and results in vascular hyper-permeability. A large amount of fluids and proteins penetrate the pulmonary clearance and alveolus from the vascular lumen, finally causing severe pulmonary edema (McDonald et al., 1999; Michel and Neal, 1999). This pulmonary edema is caused by increased pulmonary microvascular permeability and is an important pathological feature of acute lung injury induced by heatstroke. However, the mechanisms involved in this process are poorly understood.

The receptor for advanced glycation end products is a pattern-recognition receptor and a member of the immunoglobulin superfamily. The receptor for advanced glycation end products binds to multiple ligands and exerts its effects by affecting intracellular signal transduction and stimulating the secretion of cytokines. The receptor for advanced glycation end products plays an important role in diabetic complications, cardiovascular diseases, neurological diseases, tumors, among other conditions. In the vascular wall, RAGE is mainly located in the endothelial cells and smooth muscle cells to maintain the homeostasis of the human body (Mukherjee et al., 2008). While the body is subject to diseases such as inflammation and diabetes, the expression of RAGE significantly increases inflammatory cells, endothelial cells, and epithelial cells to activate and develop the inflammatory response (Herold et al., 2007; Jing et al., 2010).

In recent years, many studies have shown that the knockout of the RAGE gene or blocking RAGE with a neutralizing antibody had a protective effect and aided treatment of multiple organ dysfunction syndromes (MODS) caused by sepsis, including respiratory failure induced by acute lung injury (Bopp et al., 2008; Ramasamy et al., 2009). Although the mechanism of ALI caused by heatstroke is similar to sepsis in some respects, it has different pathogenesis. It is unclear whether RAGE is activated and mediated into the pulmonary microvascular hyper-permeability, and the mechanism involved in this process remained unintelligible. In this study, we aimed to explore the role of RAGE in the vascular barrier dysfunction induced by heat stress and the signal pathway by which RAGE is involved in the heat-induced endothelial response in both vivo and in vitro levels, finding a novel target for the clinical treatment of ALI induced by heatstroke.

## Materials and Methods

### Chemicals and Reagents

C57BL/6 wild mice of approximately 18–22 g weight were purchased from the Experimental Animal Center of Guangdong Province. RAGE null knockout mice were generated by mating homozygotes of RAGE from wild type. The primary human umbilical endothelial cell (HUVECs) line was from Sciencell. DMEM/F12 medium and fetal bovine serum (FBS) were from Hyclone (Logan, UT, United States). Trypsin, penicillin, and streptomycin were purchased from Gibco BRL (Grand Island, NY, United States). Anti-RAGE antibody (Cat. ab3611), Anti-HSF1 antibody (Cat. ab52757), and Anti-HSF1 (phospho S326) antibody (Cat. ab76076) were purchased from Abcam (United States). MAPK (Cat. 9926), Phospho-MAPK (Cat. 9910), and C-Jun Rabbit mAb (Cat. 60A8) were obtained from Cell Signaling Technology (Beverly, MA, United States). p38 inhibitor, SB203580 (Cat. S8307), JNK inhibitor, SP100625 (Cat. S5567), and ERK inhibitor (Cat. P215) were acquired from Sigma (St. Louis, MO, United States). CCK-8 was purchased from Dojindo Molecular Technologies, Inc. (Kumamoto, Japan). Rhodamine-phalloidin used to label F-actin was acquired from YEASEN Technologies, Inc. (40734ES75, Shanghai, China). The human RAGE blocking antibody was obtained from R&D systems (Cat. AF1145, Minneapolis, MN, United States) and used at 10 mg/mL, blocking over 90% of the binding.

### Animal Treatment

Animals were housed at an environmental temperature of 22 ± 2°C on a 12 h day/night cycle and allowed free access to food and water. All animal experiments were carried out in accordance with the guidelines of the Animal Care and Use Committee of Osaka University Graduate School of Medicine. For the development of the heatstroke model, mice were placed in an artificial climate chamber with an environment temperature of 37 ± 0.5°C and relative humidity of 60% ± 5%. Rectal temperature (Tc) was measured every 15 min by a mercury thermometer. Immediately after Tc reached 42.7°C, animals were taken out of the chamber and allowed to recover at room temperature (23 ± 1°C). The control group animals were executed by the same procedure as the heatstroke group except for being sham-heated at 24 ± 0.5°C.

### Analysis of BALFs

Bronchoalveolar lavage fluids (BALFs) were collected. As part of this process, 0.5 ml of PBS were injected into the lung a trachea cannula and gently aspirated. BALF samples were centrifuged at 12,000 × *g* at 4°C for 10 min to separate the cells from the supernatant. The protein concentration of supernatants was determined by a BCA assay and sediments were smeared on a glass slide, stained with Diff Quick (Dade Diagnostics, Deerfield, IL, United States), and counted under a light microscope.

### Lung Wet/Dry Weight Ratio

Mice were killed under anesthesia. The right lung was excised, weighed (wet weighed), dried in an oven at 60°C for 48 h, and re-weighed as dry weight. The wet/dry weight ratio was calculated as follows: net wet weight/net dry weight.

### Histopathology

Lung specimens from 4 mice for each group were fixed in 4% phosphate-buffered paraformaldehyde, embedded into paraffin wax, and sectioned at 5–7 μm. For histological examination, samples were stained with hematoxylin and eosin (H&E) in an automated slide Stainer as previously described. The histopathological changes of lung tissue were visualized under light microscopy.

### Blood Gas Analysis

Arterial blood was obtained via left ventricle puncture at the indicated time point, immediately taken for arterial blood gas analysis. Hydrogen ion concentration (pH), arterial oxygen tension (PaO_2_), and arterial carbon dioxide tension (PaCO_2_) were measured with a blood gas analyzer.

### Culture and Stimulations of HUVECs

HUVECs were cultured in DMEM/F12 supplemented with 10% heat-inactivated fetal bovine serum (FBS) at 37°C in a humidified atmosphere with 5% CO_2_. When reaching about 90% confluence, cells were cultured in a serum-free medium for another 12 h before it was used for the experiment.

### Cell Viability Assay

The cell viability was measured using a cell counting kit-8 (CCK-8, Dojindo Molecular Technologies, Inc., Kumamoto, Japan). Cells were planted in 96-well culture plates and treated accordingly, for different purposes. The media was then replaced with 100 μL media including 10 μL CCK-8. After 4 h, the absorbance at 450 nm was measured and the HUVECs viability was assessed directly using optical density value (OD).

### siRNA Transfection

RAGE siRNA (5′-GGAAUGGAAAGGAGACCAATT-3′), HSF1 siRNA (5′-GGAAAGUGGUC CACAUCGATT-3′), and negative control siRNA (5′-UUCUCCGAACGUGUCACGUTT-3′) were purchased from GenePharma Co., Ltd (Shanghai, China). HUVECs were transfected with siRNA using siRNA-Mate^TM^ reagent (GenePharma, China). After 4–6 h of culture, the cells were replated, cultured for additional 48–72 h, and then used for experiments.

### Adenovirus Transduction

For adenoviral transduction, HUVECs were exposed to fresh medium containing Ad-RAGE (multiplicity of infection = 200) for 1 h. The medium was then removed, and the cells were washed once with DMEM and re-cultured in normal medium for 24–48 h. The total protein was collected at 2, 4, 6, 8, and 10 days after transduction and analyzed for RAGE production by western blot.

### Measurement of Transendothelial Electrical Resistance (TEER)

The transendothelial electrical resistance of the HUVEC monolayer was determined using an electrical resistance system (EVOM; World Precision Instruments, Sarasota, FL, United States) with the STX2 electrode and EVOM^2^ meter according to the instruction manual of manufacture. HUVECs were seeded with a number of 1 × 10^5^/cm^2^ on the fibronectin-coated membrane transwell inserts (6.5 mm diameter inserts, 0.4 μm pore size; Corning, NY, United States) with 200 μL culture medium added to the apical chamber and 600 μL to the basolateral Chamber, and used until full confluence. A pair of chopstick electrodes were placed at three different points of each of the apical and basolateral chambers to evaluate TEER. Resistance values were measured every day until the TEER had risen steadily above 210 Ω⋅cm^2^ which shows that the HUVEC monolayer was fully confluent and the experiments could be carried out. TEER was measured before and after heat stress in experiments involving temperature changes.

### Endothelial Monolayer Permeability Assay

HUVEC were plated in fibronectin-coated membrane transwell units (6.5 mm diameter inserts, 0.4 μm pore size; Corning, NY, Corning, United States) and cultured to confluence in DMEM. At the start of the experiment, the culture medium in the lower and upper compartments was replaced with the new one. After incubation, 1 mg/ml of FITC-labeled dextran (initial concentration 400 mg/ml) was added to the upper compartment. After 1 h of additional incubation at 37°C, the medium in the lower compartments was collected and analyzed in a fluorescence detector using 485 and 538 nm as the excitation and emission wavelengths, respectively.

### Western Blot

Total cellular extracts were prepared by lysis and sonication of the cells in lysis buffer (20 mmol/L Tris pH 7.4, 2.5 mmol/L EDTA, 1% Triton X-100, 1% deoxycholic acid, 0.1% SDS, 100 mmol/L NaCl, 10 mmol/L NaF, 1 mmol/L Na_3_VO_4_) with protease and phosphatase inhibitors. Samples were subjected to SDS-PAGE, and proteins were transferred to polyvinylidene Fluoride (PVDF) membranes. Blots were blocked with 5% bovine serum albumin in TBS containing 0.5% Tween 20 (TBS-T) for 2 h and then incubated with a 1:1000 dilution of primary antibody for p-p38, p-ERK1/2, p-JNK or total p38, ERK1/2, JNK overnight at 4uC on a rocker. After three washes for 10 min each with TBS-T, the blots were incubated with a 1:5000 dilution of HRP-conjugated species-specific respective secondary antibody for 1 h at room temperature. After washing three times for 10 min each with TBS-T, protein bands were visualized by chemiluminescence, and then densitometric analysis was performed using a Kodak IS4000R Imaging Station.

### RT-PCR

Total RNA was extracted from the cultured cells immediately or 2 h after 2 h of heat stress at 42°C following the manufacturer’s instructions of Total RNA Kit II (Omega, United States). 1 μg RNA was reverse-transcribed to cDNA and 250 ng cDNA was amplified with primers specific for RAGE: 5′-GGGGCAG TAGTAGGTGCTCAA-3′ and 5′-GCCTGTGTTCAGTTTCCA TTC-3′; β-actin: 5′-GATGCAGAA GGAGATCACTGC-3′ and 5′-ATACTCCTGCTTGCTGATCCA-3′ using Prime Script RT-PCR Kit (Takara, China) under the following conditions: 30 cycles of 94°C for the 30 s, 55°C for 30 s, then 72°C for 1 min. β-actin was used as the endogenous reference gene to normalize the data.

### Immunofluorescent Test

The distribution of cytoskeleton F-actin in HUVECs was observed with an immunofluorescent test. Subsequently, the medium was removed and the cells were washed with PBS, permeabilized with 4% formaldehyde and 0.5% Triton X-100 at 4°C for 30 min. Cells were washed in PBS twice, then stained with rhodamine-phalloidin at 1:100 diluted for 1 h. Cells were then imaged with a Zeiss LSM780 laser confocal scanning microscope (Zeiss, Germany).

### Statistical Analysis

Data were normalized to control values and are reported as a percentage of the baseline values (mean ± SD) from at least three independent experiments. Results were analyzed by one-way ANOVA followed by post-hoc comparison. LSD post hoc analysis was used to compare data among multiple groups when equal variances assumed while Dunnett’s T3 test was adopted when not assumed. The level of significance was set at *P* < 0.05.

## Results

### Knockout of the RAGE Gene Played a Protective Role in Acute Lung Injury Induced by Heatstroke in Mice

**FIGURE 1:**
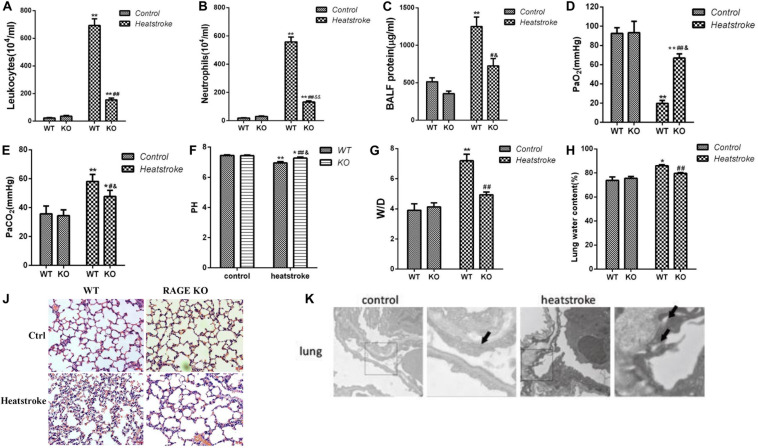
Knockout of the RAGE gene played a protective role in acute lung injury induced by heatstroke in mice. Mice were placed in an artificial climate chamber with an environment temperature of 37 ± 0.5°C and relative humidity of 60% ± 5%. Immediately after Tc reached 42.7°C, animals were taken out of the chamber and allowed to recover at room temperature for 2 h. **(A–C)** The leukocytes, neutrophils, and protein concentration in BALF, **(D,E)** Arterial blood gas parameters, **(G,H)** Lung wet/dry ratio, and water content were measured (*n* = 3, *P < 0.05, **P < 0.01 vs WT control group; #*P* < 0.05, ##*P* < 0.01 vs WT heatstroke group; ^&^*P* < 0.05, ^&&^*P* < 0.01 vs KO control group). **(J)** Histopathological changes in lung tissue for RAGE gene knockout mice suffered from heatstroke. The heat stressed mice displayed swelling of the alveolar epithelial cells, thickened alveolar wall, telangiectasia, and inflammatory cell infiltration in the alveolar interstitium. The knockout of the RAGE gene significantly attenuated histopathological changes in the lung tissue of heatstroke mice. **(K)** Morphological ultrastructure in lung tissue for mice of the control group (*n* = 3) and heatstroke group (*n* = 3). Images were obtained by electron microscopy. Arrows indicate intercellular junctions. Junctions between interconnecting cells were integral in the control group while heat exposure resulted in the disruption of intercellular junctions.

We found that knockout of the RAGE gene significantly decreased lung wet/dry weight ratio and leukocytes, neutrophils, and protein concentration in BALF (Figures 1A–H). Histopathological changes (Figure 1J) and morphological ultrastructure (Figure 1K) of lung tissue demonstrated that heat stress caused microvascular thrombi, neutrophils adhesion, and pulmonary edema, which was attenuated by knockout of the RAGE gene. These data suggested that knockout of RAGE attenuated pulmonary microvascular hyperpermeability in heatstroke mice.

### Increasing Temperature and Heat Stress Time Induces Endothelial Monolayer Barrier Disruption

Endothelial monolayer barrier integrity and paracellular permeability were determined by the measurement of TEER and flux of FITC-dextran. Since basal resistance slightly differed in independent wells, the data are presented relative (% TEER) to baseline (before heat exposure = 1).

**FIGURE 2:**
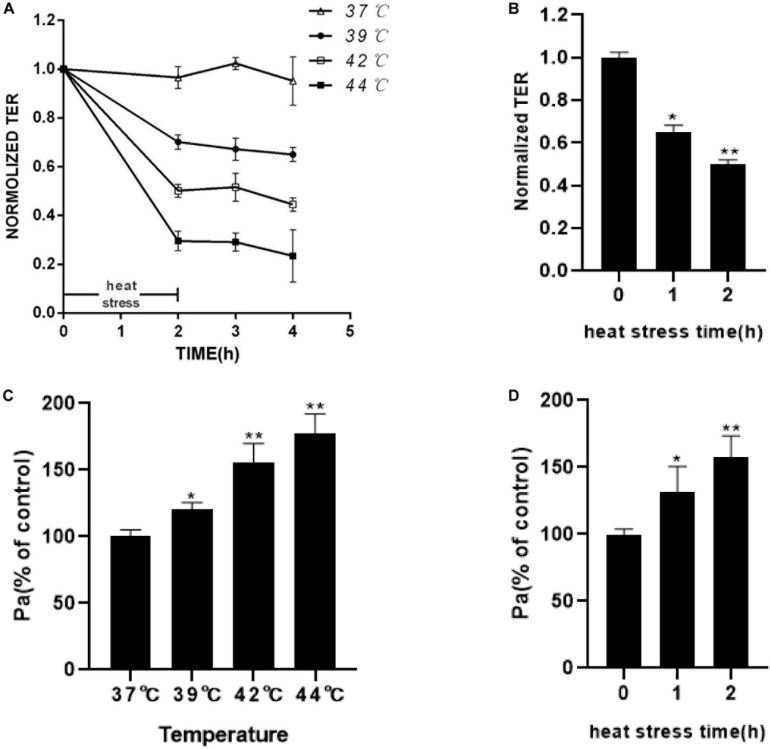
Effect of increasing temperature or increasing heat stress time on HUVEC monolayer barrier function. **(A)** Increasing temperature decreased TEER. HUVEC monolayers were exposed to an increasing temperature for 2 h from 37 to 44°C. TEER was recorded before (used as a baseline) and after heat stress and presented relative (%TEER) to baseline. **(B)** Increasing heat stress time decreased TEER. HUVEC monolayers were exposed to 42°C for an increasing time from 1 to 2 h. TEER was measured before (used as a baseline) and 2 h after heat stress and presented relative (%TEER) to baseline. **(C)** Higher temperatures increased FITC-dextran flux. The amount of dextran flux was measured 2 h after heat stress and expressed as a percentage of control. **(D)** Increasing heat stress time increased FITC-dextran flux (*P < 0.05, **P < 0.01 vs control group).

The results showed that increasing the temperature and heat stress time resulted in the reduction of TEER. In the first 2 h of heat stress, the increasing temperature showed a significant decrease in TEER in the HUVECs (Figure 2A). As shown in Figure 2B, TEER was decreased for over 2 h after heat stress, compared with that in the 37°C group. The permeability for FITC-dextran into the basolateral chambers, which was determined by the calculated flux, was expressed as a percentage of added FITC-dextran. The significant increase in paracellular permeability of FITC-dextran flux was accompanied by a reduction in TEER. Increasing temperature and heat stress time also correlated with a significant increase in FITC-dextran flux (Figures 2C,D). Next, we tested the effect of temperature on the viability of the cells by using the CCK8 assay (Supplementary Figure 1). The results showed that the viabilities of HUVECs treated with heat stress at 39 or 42°C were similar to those at 37°C. However, the viability of HUVEC at 44°C decreased significantly, which indicated that the increased permeability at 44°C may be due to the loss of cells other than the disruption of endothelial barrier function. These results indicated that increasing the temperature and heat stress time significantly weakened the endothelial monolayer barrier function related to the drop in TEER and the increase in FITC-dextran permeability.

### Increasing Temperature and Heat Stress Time Regulates Expression of RAGE Proteins

**FIGURE 3:**
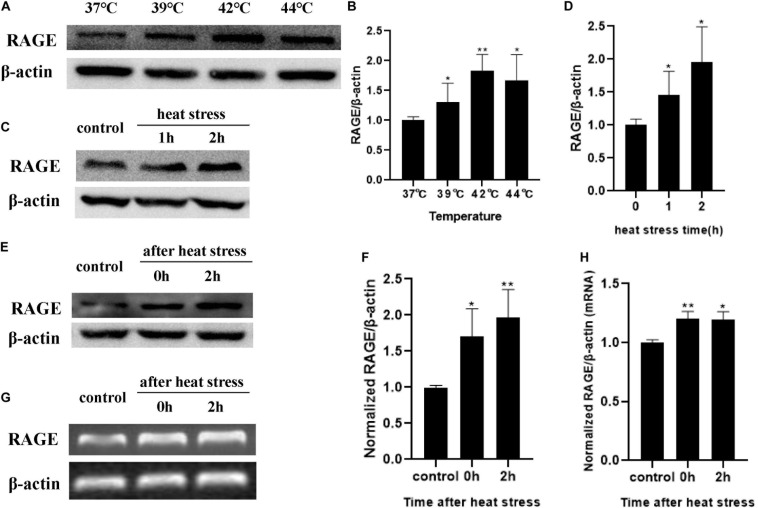
Influences of heat stress on RAGE mRNA and protein expression. HUVECs were subjected to heat stress at different conditions, and cells were harvested at a different time point after heat exposure and analyzed by western blot or RT-PCR. **(A,B)** HUVECs were subjected to heat stress at 37, 39, 42, or 44°C for 2 h. RAGE protein expression increased in a temperature-dependent manner. **(C,D)** HUVECs were exposed to 42°C for 1 or 2 h. Samples were harvested 2 h after heat stress. RAGE protein expression increased in a time-dependent manner. **(E–H)** Samples were harvested immediately or 2 h after 2 h of heat stress at 42°C and the mRNA level and protein level of RAGE increased immediately after heat stress (*P < 0.05, **P < 0.01 vs control).

Cells were exposed to designated temperatures (from 37 to 44°C) for 2 h or subjected to 42°C for increasing time (1 and 2 h). The expression of RAGE proteins was examined by Western blotting analysis. The expression of RAGE increased from 37 to 44°C and seemed to reach maximal levels at 42°C (Figures 3A,B). Exposed to 42°C for increasing time, RAGE protein expression was also significantly increased in a time-dependent manner (Figures 3C,D). We also extracted total proteins immediately or 2 h after 2 h of heat stress at 42°C, and found that RAGE protein expression was increased immediately after heat stress, and kept this effect until 4h after heat stress (Figures 3E,F). RT-PCR results showed the effects on the expression of mRNA. Heat exposure resulted in a progressive increase in RAGE mRNA expression (Figures 3G,H). These results suggested that increasing temperature and heat stress time, up-regulated the expression of RAGE proteins.

### RAGE Mediated HUVEC Monolayers Hyperpermeability Induced by Heat Stress

**FIGURE 4:**
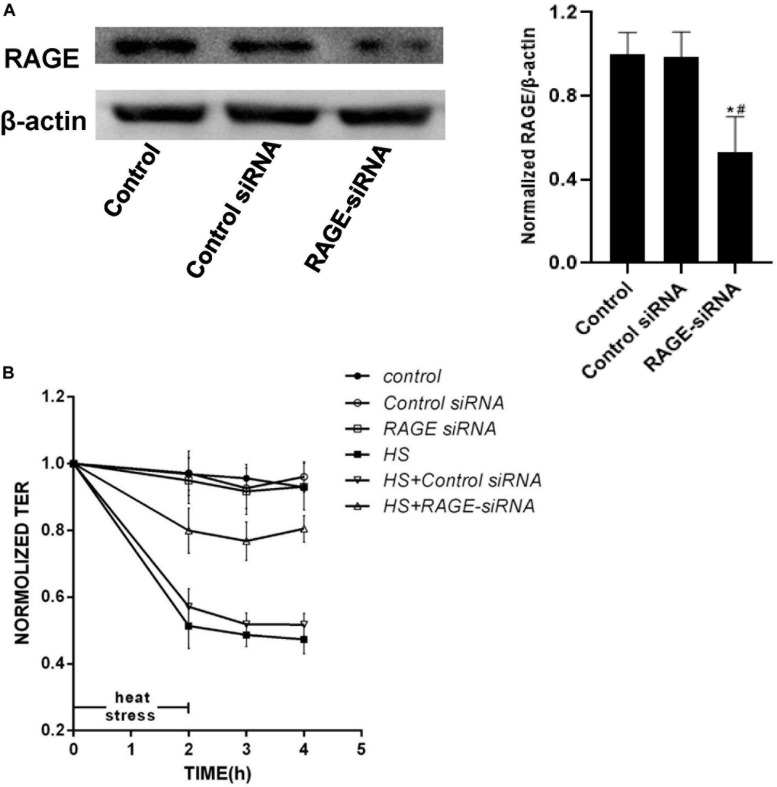
Knockdown of expression of RAGE by siRNA prevented increased permeability induced by heat stress. **(A)** HUVECs were transfected with RAGE siRNA. After 48 h, total proteins were extracted and the expression of RAGE protein was detected by western blot. **(B)** HUVECs treated with either control or RAGE siRNA were subjected to heat stress at 42°C for 2 h. TEER was recorded before (used as a baseline) and after heat Stress. Transfection with RAGE siRNA decreased the hyperpermeability induced by heat stress (*P < 0.05 vs control group, ^#^*P* < 0.05 vs control siRNA group).

To verify whether RAGE was involved in the endothelial monolayer hyper-permeability induced by heat stress, we down-regulated RAGE protein expression by transfecting siRNA, and efficiency was confirmed by WB (Figure 4A). We found that the elevation of endothelial permeability caused by heat stress was significantly inhibited by transfecting RAGE siRNA (Figure 4B). Based on these results, we concluded that heat exposure triggered HUVEC monolayer hyperpermeability by increasing RAGE protein expression.

### ERK and P38 MAPK Involved in the Signal Pathway by Which RAGE Is Involved in Heat-Induced Endothelia Barrier Dysfunction

**FIGURE 5:**
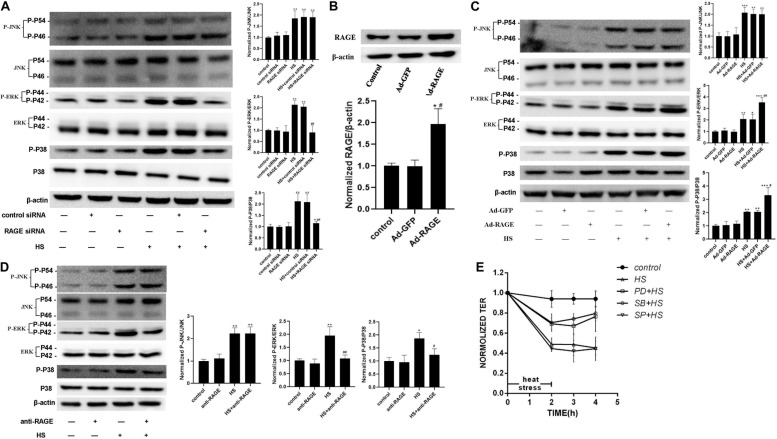
ERK and P38 MAPK involved in the signal pathway by which RAGE involved in heat-induced endothelia barrier dysfunction. **(A)** HUVECs were treated with either control or RAGE siRNA. Cells were then subjected to heat stress at 42°C for 2 h. Samples were harvested 2 h after heat exposure. The protein expression and phosphorylation of JNK, ERK, and P38 were evaluated by western blot analysis. β-actin was used as a loading control. the phospho/total MAPK is normalized to 1 in the untreated control. **(B)** Evaluation of transfection efficiency of adenovirus expressing RAGE. Cells were infected with Ad-RAGE or Ad-GFP for 48 h, and total protein was extracted and subjected to western blot to evaluate the expression of RAGE. **(C)** Cells infected with Ad-RAGE or Ad-GFP were subjected to heat stress at 42°C for 2 h. Samples were harvested 2 h after heat exposure and analyzed by western blot. **(D)** HUVEC monolayers were treated with heat at 42°C for 2 h after an absence (control) or presence of RAGE antibody for 1 h. Samples were harvested 2 h after heat exposure. The protein expression and phosphorylation of JNK, ERK, and P38 were evaluated by western blot analysis. **(E)** HUVEC monolayers were treated with heat at 42°C for 2 h after an absence (control) or presence of JNK, ERK, or P38 MAPK inhibitor for 1 h. Samples were harvested 2 h after heat exposure. TEER was recorded before (used as a baseline) and after heat Stress. Values are presented as a percentage relative to that before heat stress (*n* = 3; *P < 0.05, **P < 0.01, ***P < 0.001 vs control group, ^#^*P* < 0.05, ^##^*P* < 0.01 vs HS group).

To examine the influence of heat stress on MAPK families expression and the interaction between MAPK and RAGE after heat exposure, a series of experiments were accomplished in cells by transfecting RAGE siRNA (Figure 5A), ad-RAGE (Figures 5B,C) or pre-incubated with RAGE blocking antibody (Figure 5D). We used western blot to explore whether using antibodies specific to the kinaseshe influence of heat stress on JNK, ERK, and P38 phosphorylation. We also examined total protein expression, as well as the effect of RAGE involved in heat-induced JNK, ERK, and P38 phosphorylation. HUVECs were transfected with siRNA to decrease RAGE protein expression, pre-incubated with neutralizing antibody for 1 h to inhibit RAGE activation (Figures 5A,D), or transduced with an adenovirus over expressing RAGE gene (Figures 5B,C), then subjected to 42 for 2 h. Total protein samples were collected 2 h after heat stress. As a control, control siRNA or blank adenovirus was transfected in the same way. We found that heat stress increased the phosphorylation levels of JNK, ERK, and P38. RAGE siRNA or neutralizing antibody suppressed the phosphorylation of ERK and P38 but had no similar effect on JNK phosphorylation (Figures 5A,C). Conversely, overexpression of RAGE significantly increased the phosphorylation levels of ERK and P38 after heat stress (Figure 5B). Therefore, we can conclude that ERK and P38 MAPK are involved in heat-induced RAGE protein expression.

To further define the relations between the activation of MAPK families and the endothelial dysfunction induced by heat stress, we examined the influence of heating on HUVEC monolayer permeability by pre-treating it with specific inhibitor for 1 h to inhibit MAPK activation. As showed in Figure 5E, pre-treatment with ERK and P38 inhibitor prevented endothelial barrier dysfunction after heat stress, but a similar effect was not observed on the JNK inhibitor group. All the experiments consistently showed that ERK and P38 MAPK were involved in the signal pathway by which RAGE is involved in heat-induced endothelia barrier dysfunction. We also tested the effect of p38 on the actin cytoskeleton using Immunofluorescent assay (Supplementary Figure 2). The changes induced by heat stress included cell retraction and rounding, decreased cellular pseudopod, and stress fiber formation. Inhibition of p38 MAPK by SB203580 prevented such changes caused by heat exposure.

### Heat Stress Increased Phosphorylation Levels of HSF1 and c-Jun

**FIGURE 6:**
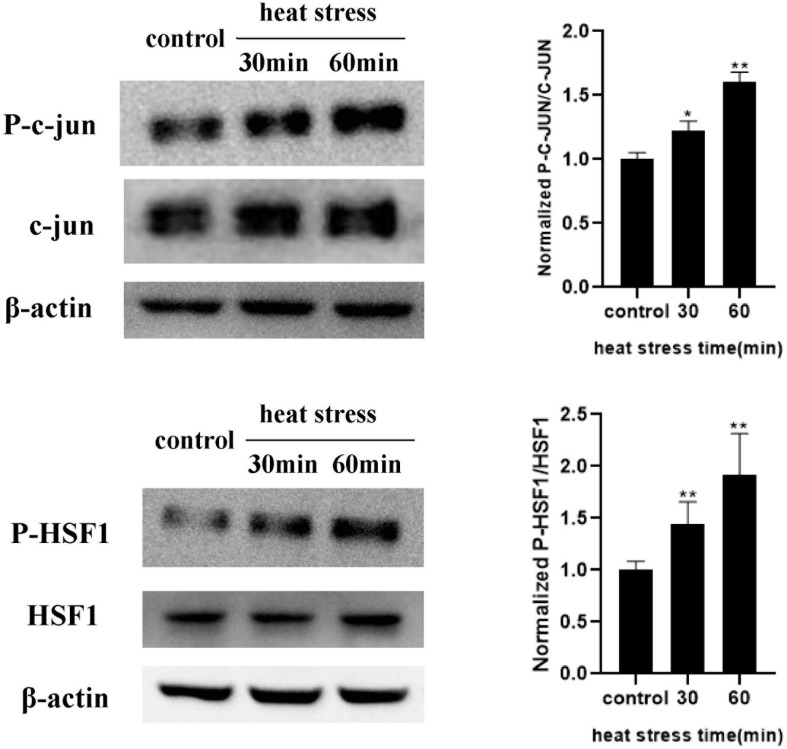
Influences of heat exposure on the protein expression and phosphorylation levels of HSF1 and c-Jun. HUVECs were subjected to heat stress at 42°C for 30 min or 1 h, and HSF1 and c-Jun phosphorylation post heat stress were determined by western blot. Representative images of western blot and quantitative analysis of phosphorylation levels of HSF1 and c-Jun normalized to total c-Jun were shown above (*P < 0.05 vs control, **P < 0.01 vs control).

To further explore the transcriptional regulation of the RAGE gene under heat stress, we analyzed the promoter region of the RAGE gene to identify the transcription factor-binding sites using the bioinformatics program TF search. We found that c-Jun and HSF1 were likely to bind to the promoter region of the RAGE gene to regulate its expression. We examined the protein expression and phosphorylation levels of HSF1 and c-Jun immediately after heat stress at 42°C for 30 min and 1 h (Figure 6). The results indicated that the phosphorylation levels of HSF1 and c-Jun increased immediately after heating for 30 min, and seemed to further elevate when prolonging heat stress time.

### Involvement of HSF1 and c-Jun in Heat-Induced RAGE Expression Changes

**FIGURE 7:**
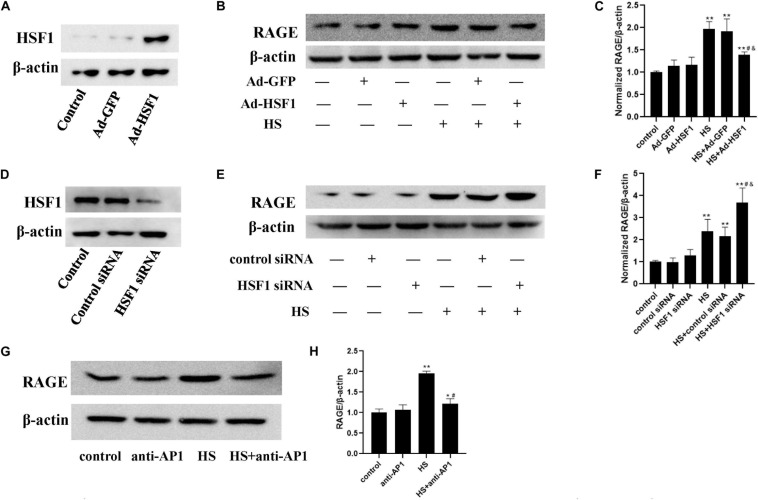
Involvement of HSF1 and c-Jun in heat-induced RAGE protein expression changes. **(A,D)** Evaluation of transfection efficiency of HSF1 adenovirus or siRNA. Cells were infected with HSF1 adenovirus or siRNA for 48 h, and total protein was extracted and subjected to western blot to evaluate the expression of HSF1. HUVECs were pre-transfected with HSF1 adenovirus to overexpress or HSF1 siRNA to downregulate HSF1, or incubated with AP-1 inhibitor to inhibit the function of c-Jun, followed by heat stress at 42°C for 2 h, and the RAGE protein expression was detected 2 h after heat stress by western blot. **(B,C)** Overexpression of HSF1 using adenovirus vector suppressed the expression of RAGE protein after heat stress; **(E,F)** Knockdown of HSF1 using siRNA significantly increased the expression of RAGE protein after heat stress. **(G,H)** AP-1 inhibitor suppressed heat-evoked elevation of RAGE protein expression (*P < 0.05, **P < 0.01 vs control group, ^#^*P* < 0.05 vs HS, ^&^*P* < 0.05 vs HS+Ad-GFP/control siRNA group).

To determine the influence of HSF1 activation to the expression of RAGE upon heat stress, we overexpressed the HSF1 gene by transducing it with an adenovirus (Figures 7A–C) or decreased HSF1 protein expression using siRNA transfection (Figures 7D–F), and then subjected it to 42°C for 2 h. Total protein samples were collected 2 h after heat stress. Control siRNA or blank adenovirus was used as a control. Finally, we found that in HSF1-overexpressing cells, RAGE protein expression was suppressed after heat stress. Conversely, RAGE siRNA significantly increased the elevation of RAGE protein expression after heat stress. These results indicate that HSF1 played a disincentive role in RAGE protein expression under heat stress conditions.

For the detection of the influence of c-Jun on heat-induced RAGE expression, a specific inhibitor, AP-1, was added 1 h before heat stress at 42°C for 2 h. The expression of RAGE 2 h after heat exposure was determined by western blot (Figures 7G,H). The inhibition of c-Jun abolished heat-evoked RAGE protein expression evaluation. The activation of c-Jun thus promoted RAGE protein expression under heat stress conditions.

## Discussion

The finds of the present study are consistent with previous studies and showed that heatstroke is involved in endothelial hyperpermeability (Sharma and Cervós-Navarro, 1990; Jeliazkova-Mecheva et al., 2006). However, the precise mechanism has not yet been discovered. Our study examined the role of RAGE in the vascular barrier dysfunction induced by heat stress and the signal pathway by which RAGE is involved in heat-induced endothelia response.

First, we established the animal model of heatstroke by using RAGE−/− mice and observed the role of RAGE in acute lung injury induced by heatstroke in mice by evaluating the leukocytes, neutrophils, and protein concentration in BALF, lung wet/dry ratio, histopathological changes and morphological ultrastructure of lung tissue and arterial blood gas analysis. The results suggested that the knockout of the RAGE gene played a protective role in acute lung injury caused by heatstroke in mice.

Studies have shown that blocking RAGE with neutralizing antibody or “sRAGE” reduced hyperglycemia-induced vascular hyper-permeability in diabetic mice (Wautier et al., 1996). It has shown the effects of RAGE on vascular hyper-permeability induced by diabetes. The inhibitor of RAGE was also reported to significantly suppress the blood-brain barrier dysfunction (Yang et al., 2015). Based on these studies, we examined the influence of heat stress on HUVEC monolayer permeability and RAGE protein expression and further explored the influence of suppression of RAGE expression by transfecting RAGE siRNA on heat-induced endothelial barrier dysfunction. Our findings suggested that heat stress increased HUVEC monolayer permeability and RAGE protein expression simultaneously in a time- and temperature-dependent manner. RAGE was essential for the heat-induced increase in HUVEC monolayer permeability.

Previous studies and our lab’s preliminary works have proved that that signaling cascades activated upon ligand–RAGE interaction, including pathways such as Erk1/2, p38, and SAPK/JNK MAPKs, rho GTPases, PI3K, and the JAK/STAT pathway. The MAPKs signaling cascades activated by RAGE can be regulated by the adaptor molecule mDia-1 (diaphanous related formin 1), a member of the GEF (guanine nucleotide exchange factor) family to activate the Rac1/Cdc42 pathway. Moreover, the RAGE cytosolic domain is connected to the tyrosine kinase protein, Src, which is also related to several downstream signal factors, such as ERK1/2, p38 MAPK, JNK (Basta, 2008; Lee et al., 2015; Zhang et al., 2015; Li et al., 2018; Riuzzi et al., 2018). To further explore the signal pathway of the heat-induced hyper-permeability in which RAGE is involved, a series of experiments were conducted, such as transfecting with siRNA, adenovirus, or pre-incubating with blocking antibody. We found that heat stress increased JNK, ERK, and P38 phosphorylation. The downregulation of RAGE diminished the phosphorylation of ERK and P38 but had no similar effect on JNK phosphorylation. Therefore, we can conclude that ERK and P38 MAPK were involved in heat-induced RAGE protein expression.

Next, we explored the transcriptional regulation of the RAGE gene under heat stress and analyzed the promoter region of the RAGE gene to identify the transcription factor-binding sites using the bioinformatics program TF search. We found that c-Jun and HSF1 were likely to bind to the promoter region of the RAGE gene to regulate its expression. Studies have shown that c-Jun is the main regulator of tumor progression in melanoma and is the most important member of the AP-1 transcription factor family in this disease (Kappelmann et al., 2014). Heat shock transcription factor 1 (HSF1) is the mammalian regulator of the heat shock response and activates the transcription of heat shock protein (Hsp) molecular chaperone genes. On the other hand, it serves as a negative regulatory role of transcription such as repression of the prointerleukin 1beta and TNFα gene (Cahill et al., 1996; Singh et al., 2000; Wang et al., 2003). There is little understanding of how they work under heat stress. The WB experiment results showed that heat stress increased phosphorylation levels of HSF1 and c-Jun. Interestingly, HSF1 and c-Jun exert an opposite function in regulating RAGE expression, among which the former suppressed RAGE expression while the latter enhanced it. These results indicate that the upregulation effect by c-Jun exceeds the downregulation effect by HSF1, which caused a gross effect of increased RAGE expression. These findings first demonstrated the following signal pathway under heat stress: C-Jun located to the promoter region of the RAGE gene and increased the RAGE protein expression after heat stress. HSF1 suppressed RAGE protein expression evoked by heat stress. The two transcription factors co-regulated the change of RAGE protein expression after heat stress, and then further increased HUVEC monolayer permeability by activating ERK and P38 MAPK. However, the specific mechanism of how heatstroke triggers the activity of c-Jun and HSF1 in facilitating RAGE expression remains unclear and will be the subject of future studies.

Overall, our findings have highlighted the critical role of RAGE in heat stress-induced endothelial hyperpermeability in acute lung injury and suggested that RAGE could be a potential therapeutic target in protecting against acute lung injury induced by heatstroke.

## Data Availability Statement

All datasets presented in this study are included in the article/Supplementary Material.

## Ethics Statement

The animal study was reviewed and approved by Animal Care Committee of the Southern Medical University of China.

## Author Contributions

LS, XHG, and QX conceived and arranged the collaboration, initiated the manuscript, edited and compiled the final version for submission. GZ, ZC, and JYL carried out most of the experimental work. GZ wrote the manuscript. XTG, KQ, JQL, and JQH helped in experimental work. QH helped in study design. All authors analyzed data, reviewed and approved the final manuscript.

## Conflict of Interest

The authors declare that the research was conducted in the absence of any commercial or financial relationships that could be construed as a potential conflict of interest.
