# Supplementary figures and images for: Role of the Receptor for Advanced Glycation End Products in Heat Stress-Induced Endothelial Hyperpermeability in Acute Lung Injury
Source: Front Physiol. 2020 Oct 7;11:1087. doi: 10.3389/fphys.2020.01087 (PMC7643755; doi:10.3389/fphys.2020.01087)

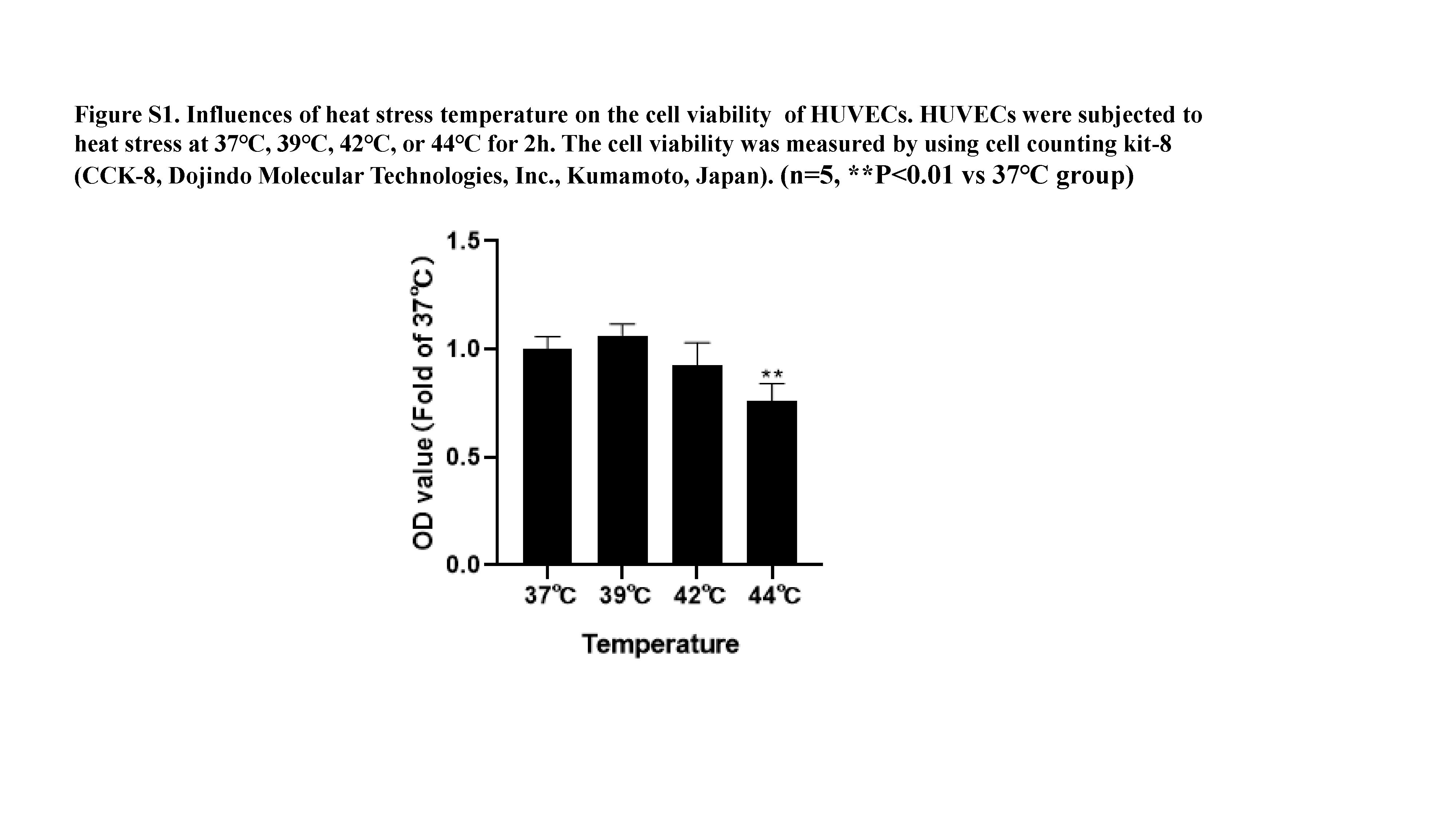

Supplement: Supplementary file 1 [file Image_1.tiff]

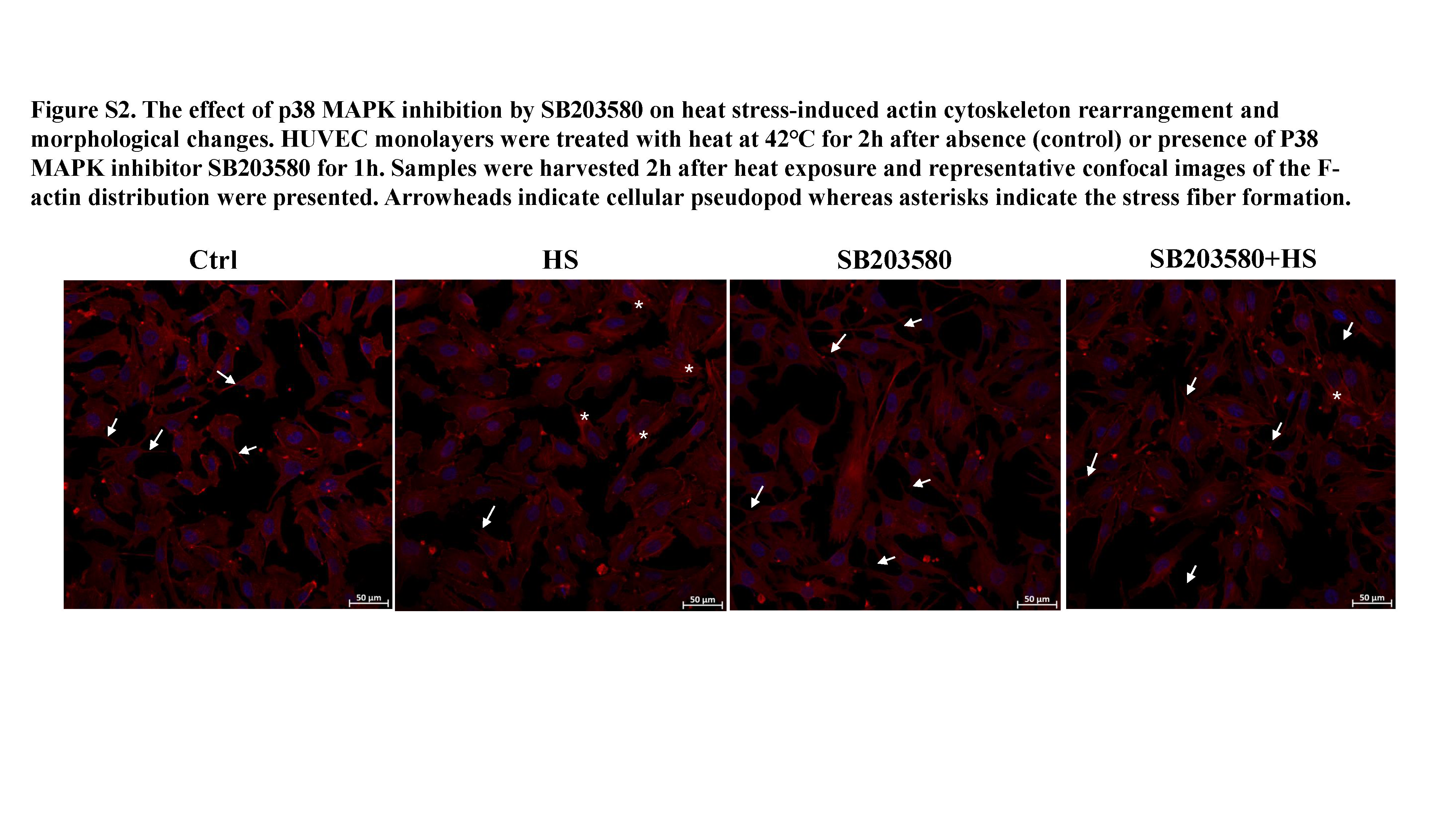

Supplement: Supplementary file 2 [file Image_2.tiff]
